# Rhodococcus equi and Arcanobacterium haemolyticum: two "coryneform" bacteria increasingly recognized as agents of human infection.

**DOI:** 10.3201/eid0302.970207

**Published:** 1997

**Authors:** R Linder

**Affiliations:** School of Health Sciences, Hunter College, New York, NY 10010, USA. rlinder@shiva.hunter.cuny.edu

## Abstract

Rhodococcus equi and Arcanobacterium haemolyticum, formerly classified in the genus Corynebacterium, are members of the loosely defined taxon "coryneform" bacteria. Although they are the etiologic agents of distinct human infections, both organisms are frequently overlooked, which results in missed or delayed diagnoses. R. equi, long known as an important pathogen of immature horses, has become in the past three decades an opportunistic pathogen of severely immunosuppressed humans. Most cases are secondary to HIV infection. When specifically sought in throat swab cultures, A. haemolyticum is found responsible for 0.5% to 2.5% of bacterial pharyngitis, especially among adolescents. These two microorganisms represent a spectrum of disease in humans: from a mild, common illness to a rare life-threatening infection. Each organism elaborates lipid hydrolyzing enzymes (cholesterol oxidase by R. equi and sphingomyelinase D by A. haemolyticum) that are toxic to animals and humans and damaging to mammalian cell membranes. The participation of the cytotoxins in pathogenicity is discussed. Greater awareness of the properties of these two bacteria may promote faster, more accurate diagnoses and better clinical management.


### *Rhodococcus equi* and *Arcanobacterium haemolyticum*: Two "Coryneform" Bacteria Increasingly Recognized as Agents of Human Infection

**Regina Linder**
Hunter College, New York, New York, USA

*Rhodococcus equi* and *Arcanobacterium haemolyticum,* formerly classified in the genus *Corynebacterium,* are members of the loosely defined taxon "coryneform" bacteria. Although they are the etiologic agents of distinct human infections, both organisms are frequently overlooked, which results in missed or delayed diagnoses. *R. equi,* long known as an important pathogen of immature horses, has become in the past three decades an opportunistic pathogen of severely immunosuppressed humans. Most cases are secondary to HIV infection. When specifically sought in throat swab cultures, *A. haemolyticum* is found responsible for 0.5% to 2.5% of bacterial pharyngitis, especially among adolescents. These two microorganisms represent a spectrum of disease in humans: from a mild, common illness to a rare life-threatening infection. Each organism elaborates lipid hydrolyzing enzymes (cholesterol oxidase by *R. equi* and sphingomyelinase D by *A. haemolyticum*) that are toxic to animals and humans and damaging to mammalian cell membranes. The participation of the cytotoxins in pathogenicity is discussed. Greater awareness of the properties of these two bacteria may promote faster, more accurate diagnoses and better clinical management.

A variety of factors contribute to the underreporting of human infections caused by bacteria in the genus *Corynebacterium* and closely related genera. The group, often referred to as "coryneform," comprises taxonomically diverse gram-positive rods resembling *Corynebacterium diphtheriae* and displaying pleomorphism and irregular cellular arrangements (1,2). The group includes human and animal pathogens, as well as commensal bacteria. The control of diphtheria in industrialized countries and the subsequent deemphasis of the genus *Corynebacterium* have contributed to discounting isolates characteristic of the genus as contaminants. Even reference laboratories report difficulty in the speciation of gram-positive pleomorphic rods that resemble corynebacteria (1). Because of the emergence of a number of coryneform bacteria as important human pathogens, rigorous biochemical and molecular tools have increasingly been applied to isolates. The resulting improved epidemiology and taxonomy have led, for example, to the definition of CDC groups JK and D-2 in the genus *Corynebacterium*, now recognized as important opportunistic pathogens (1). Similarly, more accurate characterization of some species caused them to be removed from the genus *Corynebacterium*. Excellent reviews of the pathogenicity and epidemiology of these diverse genera have been published (1,2). This article explores two pathogenic coryneform bacteria: *Rhodococcus equi*, a rare often fatal human pathogen, in which virtually all human infections occur among compromised hosts; and *Arcanobacterium haemolyticum*, which is responsible for many respiratory infections in healthy people. This article aims to bring about improved recognition of these two easily overlooked pathogens and considers mechanisms underlying the diseases, the immune response of the hosts, and treatment protocols.

Address for correspondence: Regina Linder, School of Health Sciences, Hunter College, 425 East 25th St., New York, NY 10010, USA; fax: 212-420-9135; e-mail: rlinder@shiva.hunter.cuny.edu.

### Epidemiology and Clinical Presentation

### *R. equi*

Originally isolated by Magnusson in 1923 from granulomatous lung infections in young horses (3), *Corynebacterium* (now *Rhodococcus equi*) remains an important pathogen of foals. Much of the considerable body of knowledge about *R. equi*, including its pathogenicity and immune response to infection, derives from veterinary studies and has been recently updated (4).

*R. equi* is readily found in soil, especially where domesticated livestock graze (5). The stool of horses and other animals is the source of soil contamination. Infection in humans derives from environmental exposure (2,5), and the organism may be ubiquitous in soil (6). While early cases occurred mostly in persons with a history of contact with horses, only 20% to 30% of recent cases can be traced to such contact (7). A review of cases in the three decades since the first reported human infection in 1967 is presented in Table 1.

*R. equi* is a rare opportunistic pathogen found in severely compromised patients, and most commonly in recent years, in human immunodeficiency virus (HIV)-infected persons. Early cases, most in patients receiving immunosuppressant therapy, were more likely to be successfully treated with antimicrobial agents than cases in AIDS patients (8). Most often, patients have a slowly progressive granulomatous pneumonia, with lobar infiltrates, frequently developing to cavitating lesions visible on chest x-ray. Other sites of infection include abscesses of the central nervous system, pelvis, and subcutaneous tissue, and lymphadenitis (7,8-10). Cases of lung infection caused by inhalation and cutaneous lesions caused by wound contamination have been documented; the latter are almost the only *R. equi* infections reported in healthy persons, frequently children (11). Delays in accurate diagnosis of *R. equi* are still common (2,7), despite increased awareness of this organism as an opportunistic pathogen in humans. Factors for delayed diagnosis include the insidious onset of disease, clinical resemblance of the infection to mycobacterial, fungal, and actinomycotic infections, and the relatively nondescript bacteriologic profile of *R. equi*. Morphology, partial acid fastness, and a distinctive histopathologic profile in bronchial specimens (Figure 1 A and B) contribute to accurate diagnosis. Numerous polymorphonuclear leukocytes with intracellular pleomorphic gram-positive bacteria, microabscesses, pseudotumors, and malakoplakia are noted on tissue (7,11). Malakoplakia is a relatively rare granulomatous inflammation not

Table 1. *Rhodococcus equi* case reports in humans: 1967–1996

| Years | Cases | Predisposing condition (number) | Primary site of infection (number) | Deaths | References[a] |
|---|---|---|---|---|---|
| 1967–76 | 7 | Corticosteroid (1)<br>Cancer/immunosuppressant (3)<br>Renal transplant (2)<br>None[b] (1) | Lung (6)<br>Lymphatic (1) | 0 | 8–10 |
| 1977–86 | 15 | Corticosteroid (1)<br>Cancer/immunosuppressant (4)<br>Renal transplant (2)<br>HIV (7)<br>Alcoholism (1) | Lung (14)<br>Blood (1) | 8 | 8–10,22 |
| 1987–96 | 93 | Cancer/immunosuppressant (8)<br>Renal transplant (3)<br>HIV (67)<br>Other[c] (8) (1[b])<br>None (7) (4[b]) | Lung (72)<br>Lymphatic (2)<br>Blood (8)<br>Wound (6)<br>Other[d] (5) | 34 | 8–13,22 |

[a]In the interest of space, case compilations are cited in lieu of individual case reports.
[b]Child
[c]Includes intravenous drug use, lab infection, emphysema, kidney disease.
[d]Central nervous system, gastrointestinal

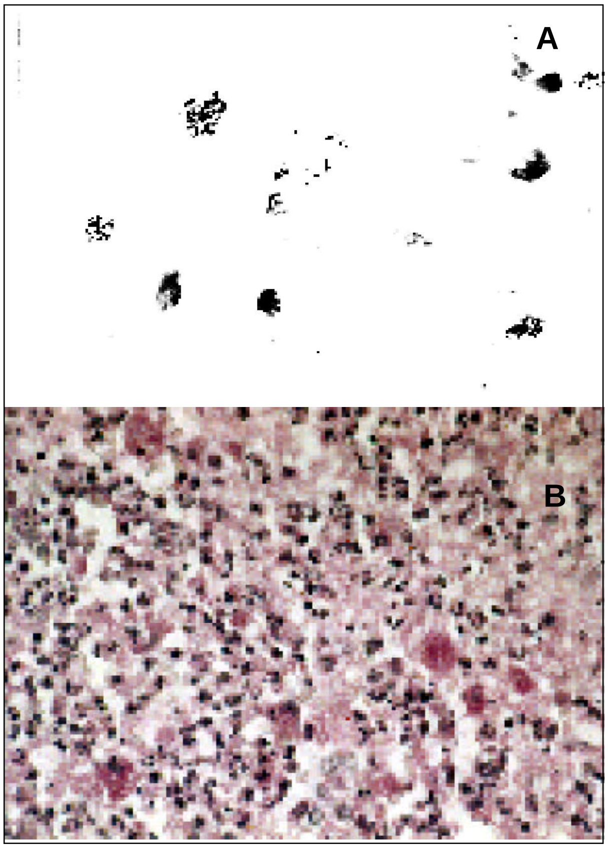

Figure 1. A. Bronchial tissue Gram stain showing intrahistiocytic coccobacillary forms of *Rhodococcus equi*. Original magnification, x 1,000. B. Open lung biopsy showing coalescent microabscesses with numerous histiocytes containing *Rhodococcus equi* organisms. PAS stain. Original magnification x 250. Figure provided by Dr. Margie Scott, Vanderbilt University Medical Center.

typically associated with histology of lung infection and can be of help in forming a differential diagnosis (11,12). Firm diagnosis and differentiation from similar pathogens require the isolation and identification of *R. equi* from sputum, bronchial washings, open-lung biopsy, or other specimens reflective of pathology. Blood cultures from severely immunosuppressed patients with focal *R. equi* infection often contain the organism. In sixty-five percent of cases secondary to HIV infection, the organism is found in patients' blood cultures (11). Deaths exceed 50% among AIDS patients with documented *R. equi* pneumonia and are almost always preceded by multiple relapses, which are common even when successful treatment is ultimately achieved (Table 1; 13).

### *Corynebacterium haemolyticum*

*A. haemolyticum* was first described and named by MacClean et al. (14), who isolated it from pharyngeal infections in U.S. soldiers and natives in the South Pacific. Classification of the organism generated controversy until the definition in 1982 of a new genus, *Arcanobacterium* (secretive bacterium), in which it remains the only species (15).

Unlike *R. equi* infection, where invasive clinical disease underscores the need to detect and identify the causative agent of infection, *A. haemolyticum* infection is often reported from deliberate screening for the organism of a large number of patients with sore throats. After it was identified during World War II from patients with pharyngitis (14), it was occasionally reported from Europe, the United States, and in 1981, Sri Lanka (16 cases) (16). Most cases involve pharyngitis and/or tonsillitis, and approximately 50% are exudative. Throat infections are often accompanied by cervical lymphadenopathy (17,18). Diagnosis of cases (distinct from screening studies) often occurs only after recurrent infections, which are thought to be related to incorrect initial diagnosis, resulting in less-than-optimum treatment (19). Infection is most common in 15- to 25-year-old persons, and is thought to result from droplet transfer from infected persons (20). Symptoms resemble those of ß-hemolytic streptococci or viral infection. The spectrum of disease ranges from sore throat to, in rare cases, a life-threatening membranous pharyngitis resembling diphtheria (18,20). An erythematous morbilliform or scarlatinal rash of the trunk, neck, or extremities is associated with 20% to 25% of cases (19), enhancing the possibility of misdiagnosis as streptococcal infection or penicillin allergy, because ß-lactam therapy is frequently initiated without accurate diagnosis. A recent report des-cribes in detail the dermatologic manifestations of *A. haemolyticum* infection (20).

The demonstration that *A. haemolyticum* is not a component of the human commensal flora was essential to establishing its role in human infection. Studies of more than 2,000 cases each found the organism only in association with clinical symptoms (17,19). Table 2 summarizes several case compilations, including the incidence of infection among culture-positive bacterial sore throats, as well as data on coinfection. Some 0.5% to 3% of cases of pharyngitis can be traced to *A. haemolyticum* depending on the population studied, with the highest numbers among 15- to

Table 2. Representative pharyngitis screenings for *Arcanobacterium haemolyticum*

| Period of study (reference) | Number of isolations | Rash | Coinfection | Incidence[a] (%) | Clinical features (population) |
|---|---|---|---|---|---|
| 1978–80 (16) | 16 | 0 | 13 (*C. diphtheriae, S. pyogenes, E. coli, P. aeruginosa*) | NR | Symptomatic throat swabs and pyoderma (Sri Lanka) |
| 1981–85 (19) | 81[b] | 37 | NR | 2.0 | Symptomatic throat swabs (Sweden) |
| 1990–92 (17) | 42 | 17 | NR | 0.36 | Symptomatic throat swabs; 5 cases monospot positive (Ottawa, Canada) |
| 1991–92 (42) | 19 | 5 | 11 (streptococci, groups A, B, G) | 0.49 | Symptomatic throat swabs, 3922[c]; (Finland) |
| 1992–95 (43) | 16 | 12 | 5 (streptococci, groups A, B, ß-hemolytic) | 0.75 | Symptomatic throat swabs, 2121[c]; (Czech Republic) |

NR-not reported
[a] Incidence refers to the proportion of sore throat cases in each study cited yielding *A. haemolyticum*.
[b] 1 of 550 asymptomatic specimens yielded *A. haemolyticum*.
[c] Figures refer to the total specimens screened.

30-year-old patients (19). Clearly, accurate diagnosis depends on differentiating *A. haemolyticum* from more common pathogens. *A. haemolyticum* occurs relatively often in polymicrobic infections together with typical respiratory pathogens such as streptococci. The isolation of classical pathogens from specimens that also contain *A. haemolyticum* exacerbates the tendency to overlook the organism.

### Taxonomy, Bacteriology, and Differential Identification

### *R. equi*

On the basis of the chain length of mycolic acids and other properties of its lipids, *R. equi* was reclassified in the suprageneric taxon nocardioform actinomycetes (1,2,5). *R. equi* is a strictly aerobic gram-positive bacterium displaying rod-to-coccus pleomorphism, with fragmenting and occasionally palisading forms. It is nonfastidious. Colonies on blood agar from clinical specimens can be mucoid and coalescing. Typical salmon pink pigmentation develops on blood agar, but often only after 2 to 3 days incubation. Growth on Lowenstein-Jensen medium allows earlier detection of pigment (M. Scott, pers. comm.). Positive routine biochemical tests include catalase and urease, but *R. equi* is generally nonreactive. Acid-fast staining of direct smears and fresh isolates is helpful in identification but is rarely observed on subculture (5,7). Some diagnostic laboratories use a commercial kit (API Coryne strip (bioMerieux-Vitek, Hazelwood, MO) for identification.

Also helpful in identifying *R. equi* is synergistic hemolysis (resembling the CAMP test), displayed by cross-streaking on sheep blood agar with any of a number of other bacteria, including *A. haemolyticum*, *Staphylococcus aureus*, and *Corynebacterium pseudotuberculosis* (21; Figure 2). Synergistic hemolysis is discussed among mechanisms of pathogenesis (below). In addition, antagonism between imipenem and other ß-lactam antibiotics used against strains of *R. equi* provides the opportunity of differentiating the organism from taxonomically related species (22).

### *A. haemolyticum*

Organisms are gram-positive rods—slender at first, sometimes clubbed, or in angular arrangements. Coccal forms predominate as the organism grows. The organism is facultatively anerobic. Growth is enhanced in blood and in the presence of $CO_2$. Some sugars are fermented, and the organism is catalase negative. Hemolysis is best observed on human blood, and Gaston et al. (20) suggest routine plating of specimens suspected of containing *A. haemolyticum* on human blood agar to distinguish *Streptococcus pyogenes*. Pitting beneath colonies on human blood agar is helpful in identification. Synergistic hemolysis with *R. equi* (or inhibition of the hemolytic zone of *S. aureus*, (Figure 2) is useful in

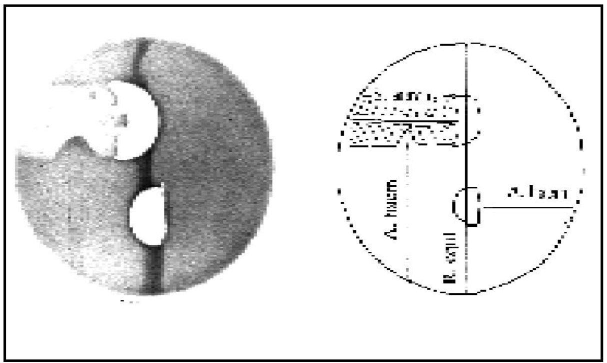

Figure 2. Cooperative (and antagonistic) hemolytic reactions on sheep blood agar, demonstrating cooperative hemolysis between *Rhodococcus equi, Arcanobacterium haemolyticum*, and *Staphylococcus aureus*. Partial hemolysis by *S. aureus* (cross-hatched on diagram) is inhibited in the proximity of *A. haemolyticum*.

identification, especially as it may rule out group B streptococci. Poor growth on tellurite assists in differentiation from *Corynebacterium diphtheriae.*

### Pathogenesis and the Immune Response

### *R. equi*

Because *R. equi* is a rare and recently emergent cause of human infection, mechanisms of its pathogenicity are not well defined. However, the much-studied infection in foals and an experimental model in mice provide data which, together with the available human data, give insight into the workings of the pathogen. Its nature as a facultatively intracellular bacterium, able to persist, grow, and ultimately destroy macrophages (23-25), is the property of *R. equi* most closely associated with virulence in each host. Foal alveolar macrophages having ingested *R. equi* did not undergo phagosome-lysosome fusion and were irreversibly damaged in electron micrographs (26). Results of pathology tests (microscopy and roentgenography) reflect significant inflammation, consistent with that found in such other intracellular pathogens as *Mycobacterium tuberculosis*, which elude pulmonary clearance. Open lung biopsies in humans show numerous polymorphonuclear leukocytes, foam cells, and cavitating lesions with intracellular bacteria (7,11; Figure 1). Also as in mycobacteria, cell wall mycolic acids are present. These acids may contribute to the ability of *R. equi* to grow in macrophages; virulence of strains for mice was found related to the carbon chain length of the molecules (27). Granuloma formation was observed when killed *R. equi* strains, regardless of virulence, were introduced into inbred mice, supporting a role for mycolic acids or other cell wall glycolipids in pulmonary inflammation (28).

A key contributor to virulence in the foal and the mouse model is a group of large (85-90 kb) plasmids, encoding 15-17 kDa antigens among strains isolated from almost all natural infections in foals (29). Strains cured of the plasmid are cleared in experimental infections, and intracellular replication in murine macrophages is greatly diminished in its absence (25,28). In contrast, of 39 strains isolated from humans (29 with AIDS), 31 strains did not express the virulence associated plasmid and were nonvirulent in mice (6). The investigators suggest that intracellular growth and, therefore, virulence among human strains may not be explained by the same determinants as foals, while mycolic acids may play a role.

Nordmann (23) found that intracellular growth in mouse and human macrophages of strains isolated from AIDS patients was related to ß-lactam resistance, the production of a bacteriophage, and virulence in inbred mice. Virulence was not attributable to 17kDa virulence antigens, but soluble cytotoxic substances were associated with the virulent phenotype. The cytotoxic activity remains to be characterized, and its relationship to known cytotoxic activities of *R. equi* remains to be elucidated. In addition to numerous hydrolytic enzymes typical of the genus *Rhodococcus* (5), strains of *R. equi*, irrespective of virulence, produce cholesterol oxidase, which is responsible for the organism's participation in synergistic hemolytic reactions with other bacteria (30; Figure 2). Experiments using cultured mouse macrophages with phagocytosed *R. equi* suggest a role for cholesterol oxidase in macrophage destruction in infections. Macrophages undergo oxidation of membrane cholesterol, and the accumulation of oxidized cholesterol is significantly enhanced by the cophagocytosis of *C. pseudotuberculosis*, a related coryneform bacterium producing sphinomyelinase D (31). Toxicity to vertebrates as a result of enzymatic oxidation of membrane cholesterol is documented in diverse systems, most dramatically by lethality to hypercholesterolemic rabbits (31).

Because *R. equi* is uniquely an opportunistic pathogen in humans, it is of interest to consider the precise nature of the immune deficiency that underlies susceptibility to this organism. Recent investigations in immunodeficient mice are especially instructive. T-cell subsets, specifically functional CD4+ lymphocytes, are necessary to effect complete clearance of *R. equi* challenge (23,32). Specifically, Kanaly et al. showed that CD4+ Th1 cells (expressing interferon-gamma) are sufficient to achieve clearance from the lungs of mice (32,33). Consistent with these data, peripheral mononuclear cells, from patients with AIDS, challenged in vitro with *R. equi* failed to secrete high levels of interferon-gamma in comparison with cells from healthy donors (34). Investigations implicating a specific defect in the Th1 phenotype in the pathogenicity of AIDS make these studies especially provocative and suggest a role for immunotherapy in *R. equi* infection (33).

While cell-mediated immunity appears to have a primary role in protecting against *R. equi* infection, the participation of humoral antibody has been established in foals. Passive immunization with hyperimmune serum is efficacious in prophylaxis, and severity of disease is inversely related to circulating antibody (1,5). Mastroianni et al. (35) demonstrated antibody to the major antigens of *R. equi* in four AIDS patients with cavitating pneumonia. The role of such antibody in the natural history of the infection remains to be elucidated.

### *A. haemolyticum*

Little is known about the mechanisms by which *A. haemolyticum* produces infection or brings about the skin manifestations frequently associated with it. The organism is known to produce uncharacterized hemolytic agent(s) (20) and two biochemically defined extracellular products: a neuraminidase and a phospholipase D (PLD) acting preferentially on sphingomyelin and generating ceramide phosphate in the target membrane (36). Of these, PLD is known to bring about tissue damage, as elaborated by this organism as well as the closely related bacterium, *C. pseudotuberculosis*, an important pathogen of sheep. Soucek et al. (36) found that the enzyme was responsible for the dermonecrotic, as well as the synergistic hemolytic, activity of the organisms that elaborate it. The PLD gene from *A. haemolyticum* has been cloned and shown to have a high degree of homology with that of *C. pseudotuberculosis*, where it is thought to participate in vascular permeability and dissemination of the pathogen (37). Evidence relates PLD with toxicity of *C. pseudotuberculosis*. Targeted mutagenesis of the PLD gene of *C. pseudotuberculosis* confirmed the role of the enzyme in virulence and specifically in dissemination in the host (38). Mutant bacteria had a reduced ability to establish infection in goats and were unable to disseminate by the lymphatics to secondary sites. A PLD sharing many properties with corynebacterial PLDs, including biochemical and biological activities, is responsible for the toxicity of the venom of the brown recluse spider (39). The role of potentiated cytotoxicity caused by the combined activity of PLD and cooperative agents such as cholesterol oxidase in disease is not established, but suggested by in vitro data involving cophagocytosis as described above (31).

### Treatment

### *R. equi*

Increased recognition of *R. equi* as a cause of life-threatening infection in severely immunocompromised persons has promoted a number of studies of in vitro antimicrobial susceptibility of clinical isolates (11,13,40,41). While variations exist, most strains were susceptible to inhibition by glycopeptide antibiotics (including vancomycin and teicoplanin) and rifampin. Macrolide antibiotics, such as erythromycin and clarithromycin, were also inhibitory to many strains. Resistance to ß-lactam antibiotics (with the exception of carbapenems, specifically imipenem) was generally reported, and is not related to the production of a ß-lactamase.

Because of relapse in spite of treatment in a majority of cases (11) and high mortality rate, especially among AIDS patients (Table 1), there is no standard treatment protocol for pulmonary and/or systemic *R. equi* infections. However, several principles reflect the accumulated experience of investigators. Careful and repeated culture and susceptibility testing during treatment is required to discover acquired resistance, in a manner similar to the treatment of mycobacterial infection (11,22). Tolerance to the cidal effects of some drugs and the need for long-term therapy (generally 2 months to life-long treatment; 40,41) make bactericidal testing a useful addition to laboratory studies. In consideration of the severe immunosuppression of patients and proclivity to relapse, investigators generally promote a combination of at least two drugs parenterally (usually including a glycopeptide or rifampin) followed by oral maintenance therapy (11,41). Recommendation of lipophilic antimicrobials that penetrate macrophages is controversial (11,41). A proposed regimen involves parenteral glycopeptide plus imipenem for at least 3 weeks, followed by an oral combination of rifampin, plus either macrolides or tetracycline (41). Examples of efficacious protocols for parenteral treatment are available (Table 1).

Surgical lung resection has been reported occasionally since the emergence of human cases, especially where large focal lesions develop (9,10), and has sometimes been efficacious in combination with antimicrobial therapy. As cases of *R. equi* continue to be recognized among AIDS patients, antimicrobial prophylaxis against this opportunistic pathogen may prove a benefit.

### *A. haemolyticum*

In vitro testing of *A. haemolyticum* isolated from human infections shows susceptibility to erythromycin, gentamicin, clindamycin, and cephalosporins (42). Reports of treatment failure with penicillin in spite of low minimum inhibitory concentrations have been attributed to tolerance and to failure to penetrate the intracellular location of the pathogen. Erythromycin has been proposed as the drug of choice, with parenteral antimicrobial drugs used for serious infections (20). The general similarity of the susceptibility pattern of *A. haemolyticum* to more commonly encountered pharyngeal pathogens, including *S. pyogenes*, makes culture and accurate diagnosis essential if cases are to be recognized for their true etiology. Its participation in polymicrobic infections (Table 2) requires that *A. haemolyticum* be specifically sought in appropriate specimens to obtain accurate diagnosis and to allow epidemiologic analysis.

*R. equi* and *A. haemolyticum* represent distinct poles of infectious disease: one a ubiquitous soil organism producing life-threatening opportunistic infections and the other a readily treatable respiratory infection of healthy young persons. In both instances, a high degree of suspicion is required to make accurate and timely diagnoses of infections. Diagnostic failure may result in a graver clinical profile including deaths for *R. equi* and, in many undiagnosed or misdiagnosed cases, for *A. haemolyticum*. As members of the morphologically defined taxon "coryneform" bacteria, these organisms exemplify properties of the group that require further elucidation. Weakly pathogenic and noninvasive, the group includes environmental bacteria; animal pathogens "crossing-over" to become human opportunistic pathogens; commensals similarly infecting compromised hosts; and producers of a wide variety of hydrolytic enzymes bearing a poorly defined relation to virulence (1). Both *R. equi* and *A. haemolyticum* elaborate a cytotoxic protein (cholesterol oxidase or sphingomyelinase D) known to be responsible for systemic harm to animals. Coincidentally, these products potentiate each other's cytotoxic action. The participation of the agents in harm to a host, alone or in combination with other substances, is consistent with available data, but yet unproven. Of particular interest is the role of cholesterol oxidase in destruction of alveolar macrophages in *R. equi* pneumonia.

Clarifying the role of synergistic or cooperative cytotoxins in one or more infectious diseases will surely improve our understanding of others because of the common occurrence of these agents among bacterial pathogens (21). It is difficult to envision the potentiated hemolytic combination of *R. equi* and *A. haemolyticum* at the site of an infectious lesion. However, cooperatively hemolytic combinations have been shown to result from the partnership of hydrolytic enzymes (e.g., phospholipases, which are ubiquitous in tissue) with the cytotoxins of pathogenic bacteria (10). Similarly, the hydrolytic enzymes of commensal bacteria or copathogens that occur, for example, in *A. haemolyticum* pharyngitis, can readily be envisioned to participate in potentiated cytotoxicity in host tissue. Together with improved recognition of these two pathogens, greater understanding of their toxic products should prove beneficial.

### Acknowledgments

I am grateful to Dr. Margie Scott for providing additional information on her cases and the micrographs in Figure 1. I thank Dr. Alan Bernheimer for his critical review of the manuscript and Dr. Patrice Nordmann for helpful discussions.

Dr. Linder is associate professor of health sciences at Hunter College, City University of New York, and director of the Medical Laboratory Sciences Program, which prepares undergraduates for careers in laboratory medicine. Her research involves the mechanisms of action and role of bacterial cytotoxins in infection.

### References

1. Coyle MB, Lipsky BA. Coryneform bacteria in infectious diseases: clinical and laboratory aspects. Clin Microbiol Rev 1990;3:227-46.
2. McNeil MM, Brown JM. The medically important aerobic actinomycetes: epidemiology and microbiology. Clin Microbiol Rev 1994;7:357-417.
3. Magnusson H. Spezifische infektioese Pneumonie beim Fohlen. Ein neuer Eitererreger beim Pferd. Archiv fur Wissenschaftliche und Praktische Tierheilkunde 1923;50:22-37.
4. Prescott JF, Holmes MA, Yager JA, Takai S, editors. *Rhodococcus equi* and immunology of the foal. Vet Microbiol. In press.
5. Prescott, JF. *Rhodococcus equi*: an animal and human pathogen. Clin Microbiol Rev 1991;4:20-34.
6. Takai S, Sasaki Y, Ikeda T, Uchida Y, Tsubaki S, Sekizaki T. Virulence of *Rhodococcus equi* isolates from patients with and without AIDS. J Clin Microbiol 1994;32:457-60.
7. Scott MA, Graham BS, Verall R, Dixon R, Schaffner W, Tham KT. *Rhodococcus equi*-an increasingly recognized opportunistic pathogen. Am J Clin Pathol 1995;103:649-55.
8. Doig C, Gill MJ, Church DL. *Rhodococcus equi*-an easily missed opportunistic pathogen. Scand J Infect Dis 1991;23:1-6.
9. Harvey RL, Sunstrum JC. *Rhodococcus equi* infection in patients with and without human immunodeficiency virus infection. Reviews of Infectious Diseases 1991;13:139-45.
10. Linder R. *Rhodococcus equi*: an emerging opportunist in humans. PHLS Microbiology Digest 1994;11:87-91.
11. Verville TD, Huycke MM, Greenfiled RA, Fine DP, Kuhls TL, Slater LN. *Rhodococcus equi* infections in humans. Medicine 1994;73:119-32.
12. Sutor GC, Fibich C, Kirschner P, Kuske M, Schmidt RE, Schedel I, Deicher H. Poststenotic cavitating pneumonia due to *Rhodococcus equi* in HIV infection. AIDS 1996;10:339-40.
13. Donisi A, Suardi MG, Casari S, Longo M, Cadeo GP, Carosi G. *Rhodococcus equi* infection in HIV-infected patients. AIDS 1996;10;359-62.
14. MacLean PD, Liebow AA, Rosenberg AA. A hemolytic corynebacterium resembling *Corynebacterium ovis* and *Corynebacterium pyogenes* in man. J Infect Dis 1946;79:69-90.
15. Collins MD, Jones D, Schofield GM. Reclassification of *Corynebacterium haemolyticum* in the genus *Arcanobacterium* gen. nov. as *Arcanobacterium haemolyticum* nom.rev.,comb.nov. Journal of General Microbiology 1982;128:1279-81.
16. Wickremesinghe RSB. *Corynebacterium haemolyticum* infections in Sri Lanka. Journal of Hygiene–Cambridge 1981;87:271-7.
17. Mackenzie A, Fuite LA, Chan FTH, King J, Allen A, MacDonald N, Diaz-Mitoma F. Incidence and pathogenicity of *Arcanobacterium haemolyticum* during a 2-year study in Ottawa. Clin Infect Dis 1995;21:177-81.
18. Green SL, LaPeter KS. Pseudodiphtheritic membranous pharyngitis caused by *Corynebacterium hemolyticum*. JAMA 1981;2330-1.
19. Banck G, Nyman M. Tonsilitis and rash associated with *Corynebacterium haemolyticum*. J Infect Dis 1986;154:1037-40.
20. Gaston DA, Zurowski SM. *Arcanobacterium haemolyticum* pharyngitis exanthem. Arch Dermatol 1996;132:61-4.
21. Fraser G. The effect on animal erythrocytes of combinations of diffusible substances produced by bacteria. J Pathol Bacteriol 1964;88:43-58.
22. Nordmann P, Nicolas MH, Gutmann L. Penicillin-binding proteins of *Rhodococcus equi*: potential role in resistance to imipenem. Antimicrob Agents Chemother 1993;37:1406-9.
23. Nordmann P, Zinzendorf N, Keller M, Lair I, Ronco E, Guenounou M. Interaction of virulent and non-virulent *Rhodococcus equi* human isolates with phagocytes, fibroblast- and epithelial-derived cells. FEMS Immunol Med Microbiol 1994;9:199-206.
24. Hietala SK, Ardans AA. Interaction of *Rhodococcus equi* with phagocytic cells from *R. equi*-exposed and non-exposed foals. Vet Microbiol 1987;14:307-20.
25. Hondalus MK, Mosser DM. Survival and replication of *Rhodococcus equi* in macrophages. Infect Immun 1994;62:4167-75.
26. Zink MC, Yager JA, Prescott JF, Fernando MA. Electron microscopic investigation of intracellular events after ingestion of *Rhodococcus equi* by foal alveolar macrophages. Vet Microbiol 1987;14:295-305.
27. Gotoh K, Mitsuyama M, Imaizumi S, Yano I. Mycolic acid-containing glycolipid as a possible virulence factor of *Rhodococcus equi* for mice. Microbiol Immunol 1991;35:175-85.
28. Takai S, Madarame H, Matsumoto C, Inoue M, Sasaki Y, Hasegawa Y, et al. Pathogenesis of *Rhodococcus equi* infection in mice: roles of virulence plasmids and granulomagenic activity of bacteria. FEMS Immunol Med Microbiol 1995;11:181-90.
29. Tkachuk-Saad O, Prescott J. *Rhodococcus equi* plasmids: isolation and partial characterization. J Clin Microbiol 1991;29:2696-2700.
30. Linder R, Bernheimer AW. Enzymatic oxidation of membrane cholesterol in relation to lysis of sheep erythrocytes by corynebacterial enzymes. Arch Biochem Biophys 1982;213:395-404.
31. Linder R, Bernheimer AW. Cytotoxicity of *Rhodococcus equi* related to generation of cholestenone in macro-phage and erythrocyte membranes. Vet Microbiol. In press.
32. Kanaly ST, Hines SA, Palmer GH. Transfer of a CD4+ Th1 cell line to nude mice effects clearance of *Rhodo-coccus equi* from the lung. Infect Immun 1996;64:1126-32.
33. Kanaly ST, Hines SA, Palmer GH. Cytokine modulation alters pulmonary clearance of *Rhodococcus equi* and development of granulomatous pneumonia. Infect Immun 1995;63:3037-41.
34. Delia S, Mastroianni CM, Lichtener M, Mengoni F, Moretti S, Vullo V. Defective production of interferon-gamma and tumour necrosis factor-alpha by AIDS mononuclear cells after *in vitro* exposure to *Rhodococcus equi*. Mediators of Inflammation 1995:4:306-9.
35. Mastroianni CM, Lichtner M, Vullo V, Delia S. Humoral immune response to *Rhodococcus equi* in AIDS patients with *R. equi* pneumonia. J Infect Dis 1994;169:1179-80.

36. Soucek A, Michalec C, Souckova A. Identification and characterization of a new enzyme of the group "phospholipase D" isolated from *Corynebacterium ovis.* Biochim Biophys Acta 1971;227:116-28.
37. Cuevas WA, Songer JG. *Arcanobacterium haemolyticum* phospholipase D is genetically and functionally similar to *Corynebacterium pseudotuberculosis* phospholipase D. Infect Immun 1993;61:4310-6.
38. McNamara PJ, Bradley GA, Songer JG. Targetted mutagenesis of the phospholipase D gene results in decreased virulence of *Corynebacterium pseudotuberculosis.* Mol Microbiol 1994;12:921-30.
39. Bernheimer AW, Campbell BJ, Forrester LJ. Comparative toxinology of *Loxosceles reclusa* and *Corynebacterium pseudotuberculosis.* Science 1985;228:590-1.
40. McNeil MM, Brown JM. Distribution and antimicrobial susceptibility of *Rhodococcus equi* from clinical specimens. Eur J Epidemiol 1992;8:437-43.
41. Nordmann P. Antimicrobial susceptibility of human isolates of *Rhodococcus equi.* Medical Microbiology Letters 1995;4:277-86.
42. Carlson P, Renoken OV, Kotainen S. *Arcanobacterium haemolyticum* and streptococcal pharyngitis. Scand J Infect Dis 1994;26:283-7.
43. Chalupa P, Jezek P, Jurankova J, Sevcikova A. Isolation of *Arcanobacterium haemolyticum* from throat culture samples in the Czech Republic. Infection 1995;23:397.
